# Art in science initiative

**DOI:** 10.1002/ansa.202000105

**Published:** 2020-10-06

**Authors:** Paul Trevorrow, Dietrich A. Volmer

## CALLING ALL ARTISTS

We tend to think of art and science as two distinct disciplines, whereas up until the 17th century the term “art” was not differentiated from the crafts or sciences.^1^ As Zhu and Goyal attest, both science and art attempt to render ideas about the world into a form that allows the viewer to connect to the idea.^2^ Both disciplines share a common goal and it is not surprising to discover the artistry underpinning some of the great scientific discoveries and creations of the modern world. From the renaissance polymath Leonardo da Vinci to Santiago Ramón y Cajal (the father of modern neuroscience) to Samuel Morse (inventor of the telegraph and Morse code) to contemporaries such as Neri Oxman (founder of the field of material ecology), the relationship between art and science is profound.^3^

“Where does the creative drive for innovative experiments come from, if not from the power of imagination?^4^”

In a series of blog articles, Marianne Dorn illustrates the overlap of art and science contrasting the scientific method with the artistic approach. Here she describes the fundamental similarities of scientific discovery—hypotheses, experiments, analysis, and presentation—with the artistic tasks of noticing, questioning, and presentation.^5^

Along Dorn's scientific career, she discovered that:

“..one day when I forgot to take off my ‘art hat’, I noticed something interesting: My grasp of new scientific ideas seemed more complete, and more satisfying, if I continued to think in an artistic frame of mind.”^5^

Dorn goes further in her analysis, providing insightful descriptions of what scientists can learn from artists. She points out that scientists would benefit from an eagerness to observe and record anything interesting at any given time and place, a readiness to seek inspiration from anywhere, a willingness to learn from emotional response without compromising objectivity, the ability to better use analogies, and the confidence to pursues things that fascinate. All these approaches are much more encouraged in art than in science.^6^

*Analytical Science Advances* (*ASA*) recognizes that as a journal we represent an extremely diverse and creative community. We also recognize our duty to contribute positively to the spirit of the community by celebrating contributors whose skills may otherwise go unnoticed in the academic structures in which they occupy. As a journal we are cognizant of our duty to nurture all strata of our community and to foster collaboration.

***Are you a researcher with a passion for art creation, cartoon drawing or illustration? Do you want to raise your profile in this arena and have your scientific artwork published?***

*ASA* invites all budding visual artists to collaborate with one of our authors on a graphic representing their work. The graphic may take the form of a graphical representation, a cartoon, infographic, or any mixed visual medium of mutual agreement. Your creation will publish alongside the research article with full authorship attribution. In addition, *ASA* will publish a showcase on you and your scientific and nonscientific artistic works. We hope that this initiative, while showcasing your artistic skills will be an opportunity to highlight your profile as a scientist, artist, and creative.

This edition of *ASA* showcases the artwork of Adrian Stavastano, In collaboration with the authors of *Influence of core size and capping ligand of gold nanoparticles on the desorption/ionization efficiency of small biomolecules in AP‐SALDI‐MS.* ^7^, ^8^

“I tremendously enjoyed speaking with Adriana about her artwork for our article. She is an artist and scientist at the same time, which made the communication smooth and easy. She captured the rather dry nature of our science in the article in her illustrations immediately. I truly believe that collaborations between scientists and artists do not only improve the communication of abstract data and theories but will also lead to new ways of representing and visualizing the data. It may sometimes yield entirely novel ways of transforming complex data sets.”

‐ Dietrich Volmer

“My gratitude to Dietrich is infinite! Working with him was my very first scientific illustration project and the first official opportunity. He trusted me completely and blindly! It would be awesome if other illustrators at “early stages” like me could have such amazing opportunities like I had with Dietrich! Thanks to him I came into contact with a research very different to mine and which I would have never had the opportunity to know about otherwise! I would say we had an instant understanding of each other's expectations for this work and I really enjoyed talking with him. Especially I liked his view of science and of doing research: he is a curious scientist, passionate and striving for learning.”

‐ Adriana Savastano

We invite budding scientific artists to take part in this initiative, who knows what doors may open for these creative endeavors!
